# Molecular mechanism of resveratrol promoting differentiation of preosteoblastic MC3T3-E1 cells based on network pharmacology and experimental validation

**DOI:** 10.1186/s12906-024-04396-3

**Published:** 2024-02-29

**Authors:** Yu He, Fei Liu, Mingjuan He, Fayu Long, Ding Hu, Jingwen Chen, Miao Fang, Zhenlong Wang

**Affiliations:** 1 Department of Spine Surgery, Chengdu Second People’s Hospital, No.2, Huatai Road, Chenghua District, Chengdu, 610000 Sichuan China; 2 Department of Spine Surgery, RuiKang Hospital affiliated to Guangxi University of Chinese Medicine, Nanning, 530011 Guangxi China; 3 Department of orthopedics, The Affiliated Traditional Chinese Medicine Hospital, Southwest Medical University, No.182, Chunhui Road, Longmatan District, Luzhou, 646000 Sichuan China

**Keywords:** Integrative medicine, Phytotherapy, Resveratrol, Apoptosis, Osteogenesis, MC3T3-E1, Osteoporosis

## Abstract

The purpose of this study was to investigate the mechanism by which resveratrol (Res) inhibits apoptosis and promotes proliferation and differentiation of pre-osteoblastic MC3T3-E1 cells, laying the groundwork for the treatment of osteoporosis (OP). The TCMSP database was used to find the gene targets for Res. The GeneCards database acquire the gene targets for OP. After discovering the potential target genes, GO, KEGG, and Reactome enrichment analysis were conducted. Verifying the major proteins involved in apoptosis can bind to Res using molecular docking. CCK8 measured the proliferative activity of mouse pre-osteoblasts in every group following Res intervention. Alkaline phosphatase staining (ALP) and alizarin red staining to measure the ability of osteogenic differentiation. RT-qPCR to determine the expression levels of Runx2 and OPG genes for osteogenic differentiation ability of cells. Western blot to measure the degree of apoptosis-related protein activity in each group following Res intervention. The biological processes investigated for GO of Res therapeutic OP involved in cytokine-mediated signaling pathway, negative regulation of apoptotic process, Aging, extrinsic apoptotic signaling pathway in absence of ligand, according to potential therapeutic target enrichment study. Apoptosis, FoxO signaling pathway, and TNF signaling pathway are the primary KEGG signaling pathways. Recactome pathways are primarily engaged in Programmed Cell Death, Apoptosis, Intrinsic Apoptotic Pathway, and Caspase activation via extrinsic apoptotic signaling pathways. This research established a new approach for Res treatment of OP by demonstrating how Res controls the apoptosis-related proteins TNF, IL6, and CASP3 to suppress osteoblast death and increase osteoclastogenesis.

**Supplementary Information:**

The online version contains supplementary material available at 10.1186/s12906-024-04396-3.

## Introduction

Osteoporosis (OP) is a metabolic bone disease that primarily affecting women after menopause and the elderly [1]. OP currently affects 200 million people globally, and the epidemic is also leading to an increase in the incidence of the disease in China. In particular, women over 40 years old are more likely than males to get OP, with a difference of 4-5 times greater than that between the sexes in this age group [2]. The primary pathological characteristics of OP are decreased bone mass and increased bone fragility, and the maintenance of bone mass and bone microarchitecture depends on the dynamic balance of osteoblast and osteoclast processes [3]. Pain, spinal abnormalities, and fragility fractures are just a few of the consequences that can result from OP [4]. The most severe complication of OP is fragility fracture, and the literature suggests that the risk of fragility fracture in female OP patients over 50 years of age may be as high as 50% [5]. As a result, OP has grown to be a significant global public health issue and places a significant financial burden on both individuals and society [6]. Understanding OP's pathogenic process and the cellular and molecular mechanisms at play is essential.

Pharmacological therapies for OP currently come in two primary categories. A bone-building agent is used to improve bone quality, while an anti-resorptive agent slows down bone loss [7]. One of the main forms of treatment for postmenopausal OP patients is estrogen replacement medication. Postmenopausal OP can be prevented and treated by using estrogen and estrogen analogues, which can directly binds to estrogen receptors in bone tissue and control bone metabolism [7, 8]. Still, continued use of these medications will likely have adverse effects, like breast cancer, heart disease, or strokes [9]. As a result, it is vital to identify a medicine that minimizes adverse effects while maintaining drug effectiveness. Chinese medicine is well known to play a significant role in the upkeep of health in Asian nations like China [10]. Complementary and alternative medicine use and increasing welcome to it by general population and diverse populations with different diseases [11–14]. Additionally, people with OP have employed herbal monomers as additional and alternative therapies [15]. Many recent studies have shown that herbs and many single components are effective in the treatment of OP, with the major mechanisms involving reduced oxidative stress, suppression of inflammatory response, and encouragement of osteoblastogenesis [16]. Herbal polysaccharides, for example, have anti-osteoporosis benefits by balancing bone resorption and bone growth [17], Icariside stimulates osteogenic differentiation of mesenchymal stem cells generated from bone marrow while suppressing osteoclast differentiation and bone resorption activity to increase bone production [18]. In the treatment of OP, traditional Chinese medicine has a distinctive and extensive body of knowledge. Due to their structural and functional similarities to human estrogen and lack of significant side effects, phytoestrogens are being used more frequently in OP application research [19]. Natural plants like thuja, grape, and peanuts contain resveratrol (Res), an ingestible phytoestrogen that has effects similar to those of mammalian estrogen [20]. Res can be used to treat OP in place of estrogen and has a bone-protective effect, according to in vitro and in vivo research [21]. Although Res is rapidly becoming known for, developed, and therapeutically used in the treatment of OP with good success, it is unclear how it actually works to counteract OP.

A component of bioinformatics is network pharmacology, which uses a priori analysis to examine the connections between medications, substances, disorders, and targets [22]. Network pharmacology is frequently utilized to clarify the mechanism of action of Chinese medicine since it has the ability to analyze multiple components, multiple targets, and several pathways, giving researchers new paths and tactics [23]. We will use a network pharmacology approach in this study to identify the key genes and pathways of Res for the treatment of OP, as well as further investigate the mechanisms associated with Res to promote proliferation and differentiation of pro-osteoblasts for the treatment of OP via Res intervention in MC3T3-E1 proliferation and differentiation.

## Materials and methods

### Bioinformatic data analysis of Res treatment OP

#### Acquisition of Res prediction targets

The Traditional Chinese Medicine Systematic Pharmacology Database (TCMSP, http://lsp.nwu.edu.cn/tcmp.php) was used to compile information on the chemical constituents and targets of action associated to Res [24]. Standardize the targets' "protein names" to their official names using the Uniprot database (http://www.uniprot.org/) [25].

#### Acquisition of Res therapeutic op action targets

Screening for disease targets with the human genetic database GeneCards (https://www.genecards.org/) [26], to find the appropriate targets for OP, enter the keyword "osteoporosis." Potential therapeutic targets for Res treatment of OP were determined by plotting the anticipated targets in "1.1" against the OP-related targets in the Venn diagram.

#### Construction and analysis of PPI network

The STRING database (https://stringdb.org) was used in this study to examine the protein interaction network of prospective treatment targets for Res on OP [27]. Cytoscape 3.9.1 software (https://cytoscape.org/) was used to visualize and analyze the data after the prospective therapeutic targets were loaded into the STRING database, the species was set to human, and a moderate interaction value of "0.4" was taken to obtain protein interactions [28]. The program was configured to represent the change in Degree size by setting the node size and color to reflect the topological qualities of possible therapeutic targets. The more nodes and the darker the Degree value, the higher the Degree value. To obtain the primary target points, filter the target points greater than or equal to one-half of the Degree value [27].

#### Drug-target-disease network construction

Using Cytoscape 3.9.1 software, combine the drug "Res" with the "potential therapeutic target" and illness name "OP" discovered in "1.2" to produce a "drug-target-disease network diagram" [29].

#### Biological processes and pathway enrichment

Import the potential target genes of Res therapeutic OP into the Bioeasy Cloud Platform, then select Enrichment Analysis in the Tool Center and limit the species to "H. sapiens". In the shared parameters, enter the gene ID of the core target and submit. Finally, we obtained the enrichment analysis results of GO, KEGG, and Reactome for the core targets of Res therapeutic OP. The final outcomes are visually represented [30–33].

#### Molecular docking to validate key target binding capabilities

Obtain the 3D structure of the proposed docking target in mol2 format from the Pubchem database, open the small ligand molecule with AutoDock 4.2 (https://autodocksuite.scripps.edu/adt/) [34], hydrogenate, charge, detect the ligand root, search and define the rotatable bond, and then save it as a pdbqt file [35]. Download the core 3D structure of the target protein from the RCSB protein database (www .rcsb.org/) as a docking protein. Add all hydrogen atoms in Autodock to open, calculate Gasteiger charge, bind non-polar hydrogen, define as receptor, and save as pdbqt file [36]. Determine the coordinates and box size of Vina molecular docking, set the parameter exhaustiveness to 15, and take the default value for other parameters. Autodock vina (https://vina.scripps.edu/) was used for semi-flexible docking [37], and the conformation with the best affinity was selected as the final docked conformation.

### Res suppression of apoptosis promotes proliferation and differentiation into MC3T3-E1 cells in Vitro

#### Experimental cell and reagent selection

Mouse pre-cranial osteoblast subclone 14 (MC3T3-E1 Subclone 14) was purchased from the cell bank of Chinese Academy of Sciences. Res was purchased from Aladdin Reagent Company, China, HPLC grade (≥ 94%). α-MEM medium was purchased from HyClone, USA. Fetal bovine serum (FBS) was purchased from Gibco, USA. TNF-a, CASP 3, IL-6 antibodies were purchased from Proteintech. CCK-8 kit (cell proliferation and toxicity assay kit) was purchased from Beijing Solabao Technology Co. Ltd.

#### Experimental method

##### Preparation of Res solution

Ten mg Res was dissolved in 438 μl DMSO to form a 100 mmol/L Res stock solution, which was split and refrigerated at -20 °C. The Res stock solution was diluted with α-MEM medium (including 10% fetal bovine serum by volume) to the following concentrations: 0.01, 0.1, 1, 10, 100 mol/L.

##### Cell culture

MC3T3-E1 cells were inoculated in α-MEM medium (containing 10% fetal bovine serum by volume) and cultured at 37 °C in an incubator with 5% CO_2_ by volume before being digested and passaged when the cells reached the logarithmic phase. When the cell growth fusion rate reached 80%, the cells were passaged once and the third and fourth generation cells were used for the experiment.

##### CCK-8 detection

MC3T3-E1 cells were grown to 80% fusion, digested with digestion solution containing 0.25% trypsin, made into cell suspension, and inoculated with 3000 cells/well in a 96-well plate and cultured at 37 ℃ in a 5% CO_2_ incubator. After 24 h of cell wall attachment, 1 μg/ml of lipopolysaccharide (LPS) was added to stimulate the cells for 24 h [38, 39]. After 24 h of cell wall attachment, 200 μL of α-MEM culture medium containing different concentrations of Res (0, 0.01, 0.1, 1, 10, 100 μmol/L, respectively) was replaced, and the cells were incubated for 24 h and 48 h. After 20 μL of CCK-8 solution was added to each well, the cells were incubated for 2 h in the incubator, and the OD values at 450 nm were read with a multifunctional enzyme marker. The OD value at 450 nm was read by multifunctional enzyme marker.

##### Alkaline phosphatase staining (ALP staining)

MC3T3-E1 cells were blown and mixed, and the plates were evenly spread with 12-well plates set at 5×10^4^ cells/well, and after 24 hours of LSP intervention, they were divided into blank control groups and 0.01, 0.1, 1, 10, and 100 μmol/L Res groups. After intervention with Res of different concentrations for a period of time, each group was replaced with osteogenic induction solution containing 50 mg/L ascorbic acid and 10 mol/L sodium β-glycerophosphate for a period of time. After observing the cell status, the medium was aspirated, the cells were washed twice with PBS, 4% paraformaldehyde was soaked for 30 min to fix the cell morphology, PBS was washed twice to remove paraformaldehyde, NBT-BCIP staining solution was added to stain the cells for 30 min at 37 ℃ to avoid light, and the stained cells were rinsed 3 times with distilled water and photographed under microscopic observation.

##### Alizarin red staining

MC3T3-E1 cells were blown and mixed, and the plates were evenly spread with 12-well plates set at 5×10^4^ cells/well, and after 24 hours of LSP intervention, they were divided into blank control groups and 0.01, 0.1, 1, 10, and 100 μmol/L Res groups. After intervention with Res of different concentrations for a period of time, each group was replaced with osteogenic induction solution containing 50 mg/L ascorbic acid and 10 mol/L sodium β-glycerophosphate for a period of time. After observing the cell status, the medium was aspirated and the cells were washed twice with PBS, and the cells were fixed by soaking in 4% paraformaldehyde for 30 min. The cells were stained with 0.2% volume fraction of alizarin red staining solution for 30 min at room temperature. After staining, the cells were washed 3 times with distilled water. The cells were observed for the presence of orange-red precipitates and mineralized nodules under the microscope.

##### Expression of bone development genes (qt-PCR)

For 24 hours following LPS stimulation, MC3T3-E1 cells were grown in blank control, 0.01, 0.1, 1, 10, and 100 mol/L Res groups to examine the expression of pre-developmental-specific genes in osteoblasts. Phosphate-buffered saline (PBS) solution was used to wash the cells twice after they had been isolated using 0.25% trypsin-edta. Cultured cells were treated with 1 mL of TRIZOL reagent to extract the total RNA (Invitrogen, USA). According to the manufacturer's instructions, a rigorous protocol was followed. UV spectrophotometry at A260/A280 was used to quantify total RNA. In the presence of reverse transcriptase, cDNA was produced after 30 minutes at 55°C, 5 minutes at 85°C, and 10 minutes at 4°C. The following cycling settings were used for qPCR on an ABIStepPnePlus machine (Thermo Fisher Scientific, USA): 50°C for 2 minutes, 95°C for 10 minutes, 95°C for 15 seconds, and 60°C for 1 minute (40 cycles). The RUNX2 and OPG primer sequences for PCR are shown in the Supplementary Material (Supplementary Table S8). All gene expression levels were normalized by GAPDH gene expression.

##### Screening Res optimal concentration to validate related proteins

After LPS pretreatment for 24 h, CCK8 assay was performed to obtain the optimal concentration of Res, and then divided into blank control group and Res optimal concentration group to verify the apoptosis-related proteins TNF-a, CASP 3, and IL-6. MC3T3-E1 cells were blown and mixed, and 6-well plates were set at 1×10^5^ cells/well to spread the plates evenly, and after LPS pretreatment for 24 h, they were divided into blank control group and Res optimal concentration group. After 24 hours of LPS pretreatment, the cells were divided into blank control group and Res optimal concentration group. After 48 hours of incubation, MC3T3-E1 cells were washed twice with PBS after the intervention, and the cells were lysed with RIPA lysis buffer to extract proteins. The proteins were separated by SDS-PAGE, transferred to PVDF membranes (after the PVDF membrane was cut into the corresponding width, it was activated and placed in the target protein position on the gel for transfer), and incubated with the corresponding primary and secondary antibodies for detection. The antibodies used included TNF-α, CASP 3, IL-6, primary antibodies and corresponding secondary antibodies. The membranes were developed in a chemiluminescence imaging system, and the results were analyzed using ImageJ software to calculate the grayscale values of each band.

### Statistical analysis

SPSS 20.0 software was used for statistical analysis, and data are presented as histograms of means ± standard error of the mean values for data from three or more independent experiments. The means between two groups were compared by independent samples t-test, and the means between multiple groups were compared by one-way ANOVA, and further two-by-two comparisons were performed by LSD test. All statistical analysis results were considered significant at *P*<0.05.

## Results

### Analysis of target data results of Res and OP

**Fig. 1:**
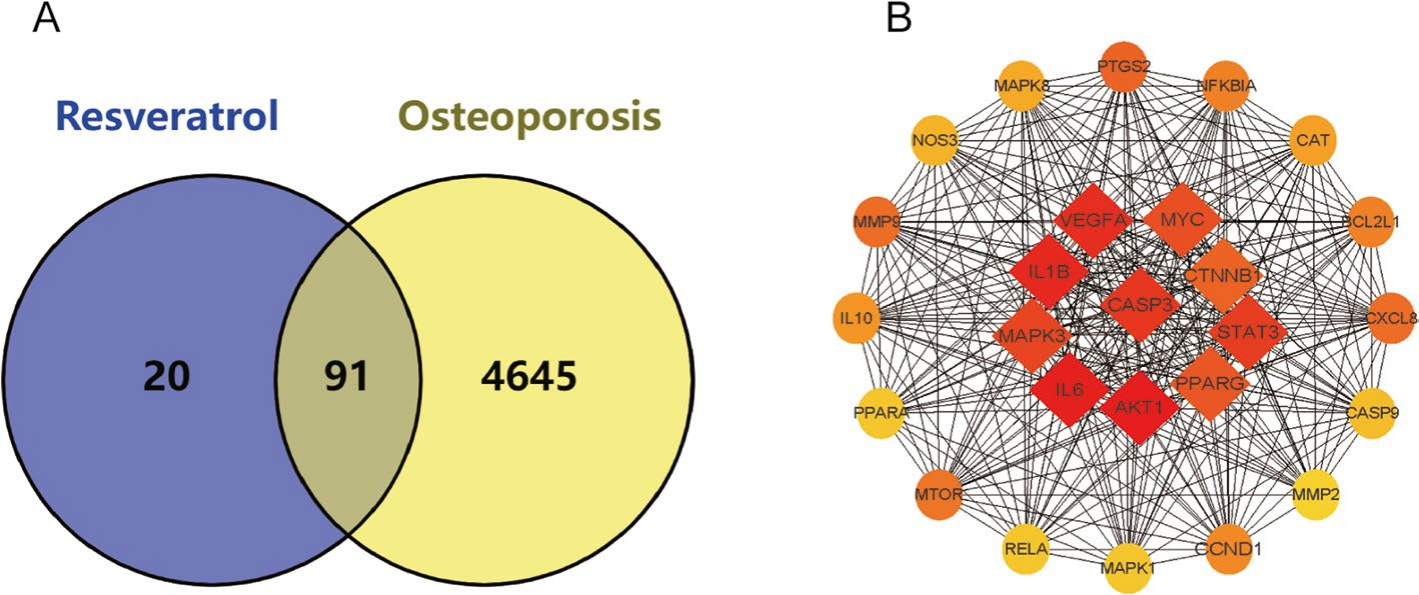
**A** Potential targets of action for research and therapy Venn diagram, **B** The top 10 important genes are among the 26 major targets of action for Res treatment of OP identified from 91 potential therapeutic targets utilizing the CytoHubba screen

Through TCMSP database screening and Uniprot database correction, we finally obtained 111 valid targets for Res (Supplementary. Table S1), and based on GeneCards database, we obtained 4376 action targets for OP (Supplementary. Table S2). Online Venn diagram analysis obtained 91 intersection targets of Res and OP, which are the potential targets of action of Res for treating OP (Fig. 1A, Supplementary. Table S3).

**Fig. 2:**
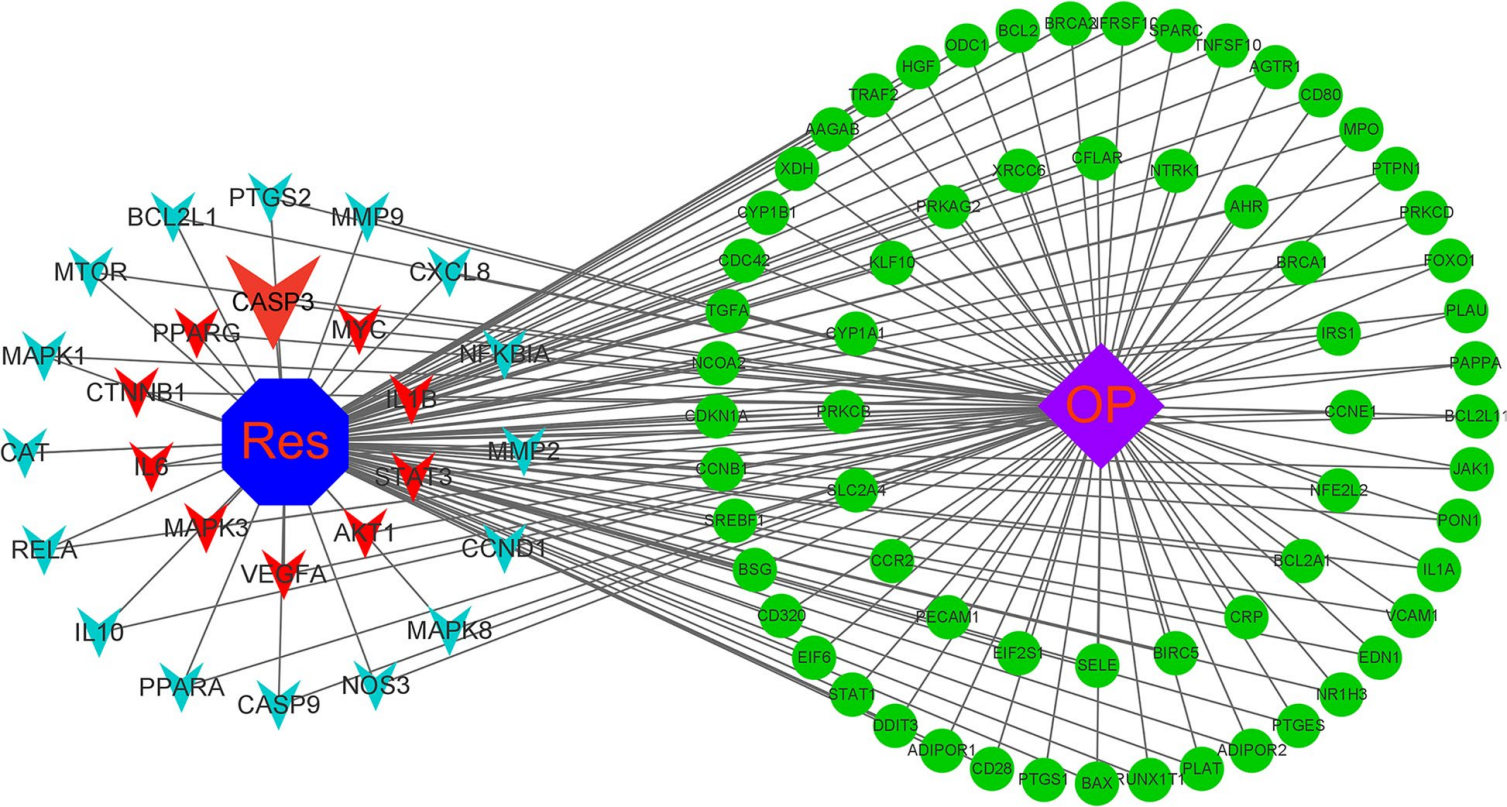
"Res-intersection gene-OP" effect network map, this figure clearly shows the potential targets for Res treatment of OP, and the key targets

In order to obtain the network interaction protein map of Res treatment OP, the 91 intersecting genes were put into the string online data analysis tool. The protein interactions data were once more uploaded into Cytoscope 3.9.1 where CytoHubba determined 26 major role targets of Res treatment OP, of which the top 10 critical genes AKT1, IL6, IL1B, VEGFA, CASP3, STAT3, MAPK3, MYC, PPARG, CTNNB1 played the key part (Fig. 1B and Table 1). After importing the Res-intersection target-OP data into Cytoscope software, we finally constructed the "Res-intersection gene-OP" effect network map (Fig. 2). Where, the nodes represent drugs, potential therapeutic targets, and diseases, and the edges represent the interrelationships between drugs and targets, and targets and diseases. Combined with the PPI analysis, the left target indicates the core target of the potential therapeutic target for Res treatment of OP. The red core target suggests an important role in Res treatment of OP.

**Table 1:** Basic information of some key targets of Res against OP

UniProt ID	Gene symbol	Protein names	Degree
P31749	AKT1	RAC-alpha serine/threonine-protein kinase	75
P05231	IL6	Interleukin-6	71
P01584	IL1B	Interleukin-1 beta	66
P15692	VEGFA	Vascular endothelial growth factor A	64
P42574	CASP3	Caspase-3	63
P40763	STAT3	Signal transducer and activator of transcription 3	62
Q16644	MAPK3	Mitogen-activated protein kinase 3	60
P01106	MYC	Myc proto-oncogene protein	58
P37231	PPARG	Peroxisome proliferator-activated receptor gamma	57
P35222	CTNNB1	Catenin beta-1	54
P35354	PTGS2	Prostaglandin G/H synthase 2	54
P14780	MMP9	Matrix metalloproteinase-9	52
P10145	CXCL8	C-X-C motif chemokine 8	52
P42345	MTOR	Serine/threonine-protein kinase mTOR	51
Q07817	BCL2L1	Bcl-2-like protein 1	50
P25963	NFKBIA	NF-kappa-B inhibitor alpha	50
P24385	CCND1	G1/S-specific cyclin-D1	49
P22301	IL10	Interleukin-10	48
P04040	CAT	Catalase	47
P45983	MAPK8	Mitogen-activated protein kinase 8	45
P29474	NOS3	Nitric oxide synthase, endothelial	43
P55211	CASP9	Caspase-9	42
Q04206	RELA	Transcription factor p65	41
P28482	MAPK1	Mitogen-activated protein kinase 1	41
Q07869	PPARA	Peroxisome proliferator-activated receptor alpha	41

### Results of enrichment analysis of res treatment OP

**Fig. 3:**
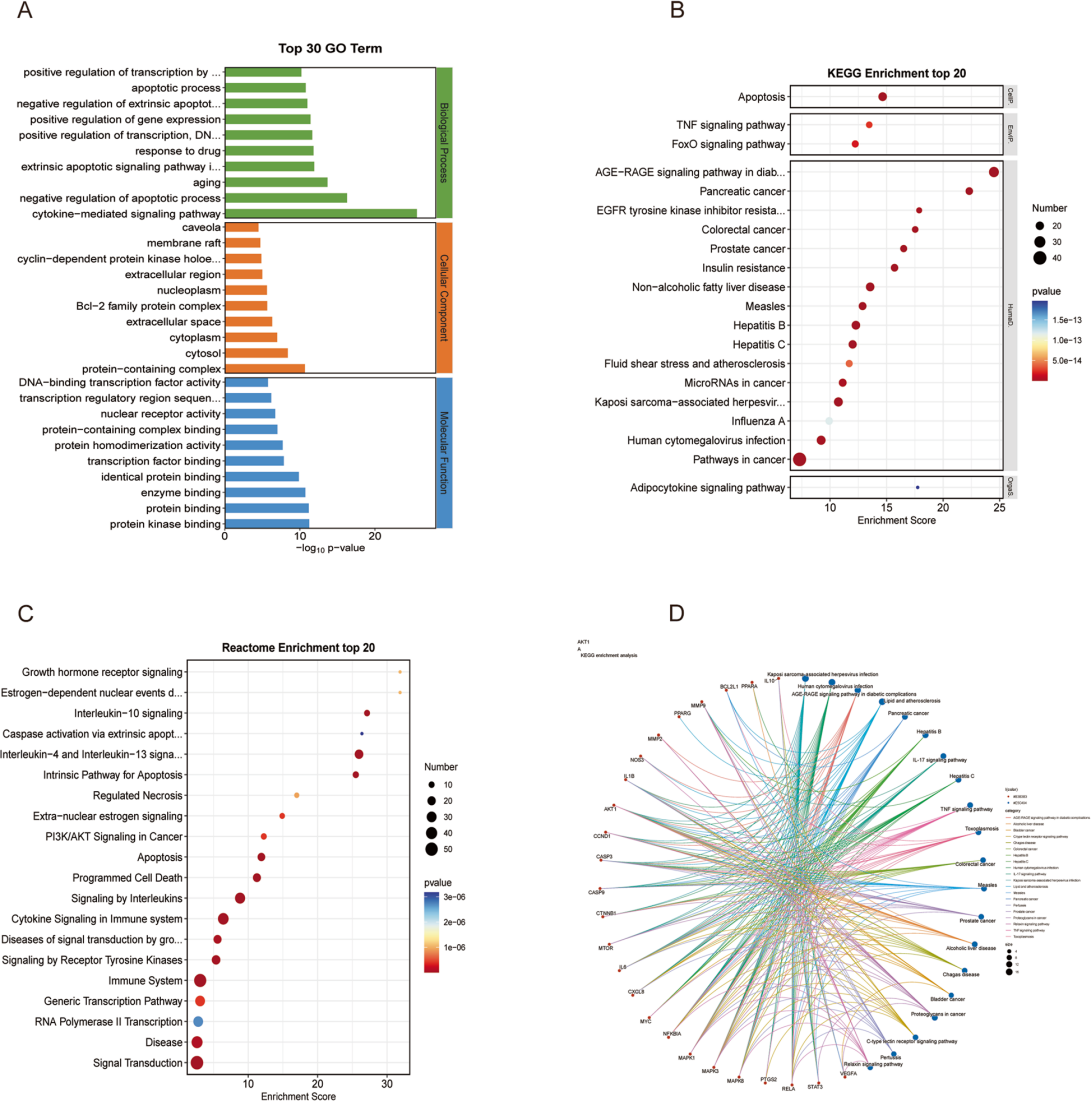
**A** GO enrichment analysis results, **B** KEGG enrichment analysis results, **C** Reactome enrichment analysis results, **D** key gene-signaling pathway correlation analysis.

**Fig. 4:**
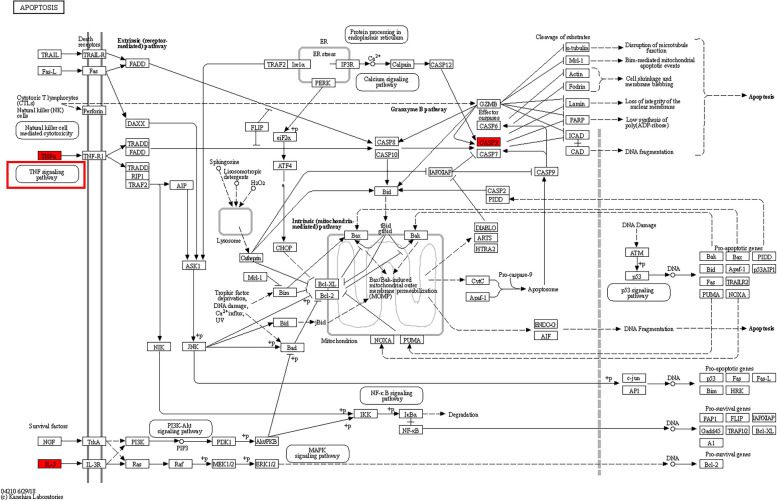
KEGG's diagram of the apoptotic signaling pathway, as shown in the figure, the TNF signaling pathway and TNF-a protein play an important role in the activation of apoptosis as the upstream pathway of apoptosis signaling

We used 91 potential targets of action to carry out GO, Reactome, and KEGG database enrichment analysis in order to clarify the biological mechanism of Res therapy of OP. By calculating the ranking through *p*-value values, we finally take the top 10 entries of BP, CC, and MF (Table 2 Supplementary. Table S4). We conclude that the biological process of Res treatment OP mainly involved in cytokine-mediated signaling pathway, negative regulation of apoptotic process, Aging, extrinsic apoptotic signaling pathway in absence of ligand, response to drug, positive regulation of transcription, DNA-templated, positive regulation of gene expression, negative regulation of extrinsic apoptotic signaling pathway via death domain 、receptors、apoptotic process, positive regulation of transcription by RNA polymerase II. Molecular function is mainly concerned with protein kinase binding, protein binding, enzyme binding, identical protein binding, transcription factor binding, protein homodimerization activity, protein-containing complex binding, nuclear receptor activity, transcription regulatory region sequence-specific DNA binding, DNA-binding transcription factor activity. Cellular component is mainly concerned with protein-containing complex, Cytosol, Cytoplasm, extracellular space, Bcl-2 family protein complex, Nucleoplasm, extracellular region, cyclin-dependent protein kinase holoenzyme complex, membrane raft, caveola (Fig. 3A). A total of 219 relevant pathways were obtained from the KEGG data analysis (Supplementary. Table S5), and the top 20 pathways with the highest association were ranked by *p*-value calculation, which were AGE-RAGE signaling pathway in diabetic complications, Pathways in cancer, Pancreatic cancer, Apoptosis, Non-alcoholic fatty liver disease, Hepatitis B, Kaposi sarcoma-associated herpesvirus infection, Insulin resistance, Hepatitis C, Prostate cancer, Measles, Colorectal cancer, Human cytomegalovirus infection, EGFR tyrosine kinase inhibitor resistance, MicroRNAs in cancer, FoxO signaling pathway, TNF signaling pathway, Fluid shear stress and atherosclerosis, Influenza A, Adipocytokine signaling pathway (Fig. 3B, Table 3). Reactome data analysis yielded a total of 791 correlated pathways (Supplementary. Table S6), and the top 20 pathways with the highest correlation were obtained by *p*-value calculation and ranking, which are areInterleukin-4 and Interleukin-13 signaling, Signaling by Interleukins, Cytokine Signaling in Immune system, Immune System, Programmed Cell Death, Apoptosis, Signal Transduction, Intrinsic Pathway for Apoptosis, Interleukin-10 signaling, Signaling by Receptor Tyrosine Kinases, Diseases of signal transduction by growth factor receptors and second messengers Disease, Generic Transcription Pathway, PI3K/AKT Signaling in Cancer, Extra-nuclear estrogen signaling, Regulated Necrosis, Estrogen-dependent nuclear events downstream of ESR-membrane signaling, Growth hormone receptor signaling, RNA Polymerase II Transcription, Caspase activation via extrinsic apoptotic signaling pathway (Fig. 3C, Table 4). Finally, we also suggested that the expression regulation of the pivotal gene was closely related to the TNF signaling pathway, the upstream signal of apoptosis signaling, based on the key gene-signaling pathway correlation analysis (Fig. 3D). All the above enrichment analysis results suggest that Res treatment OP is closely related to the apoptotic signaling pathway, and by querying the KEGG signaling pathway expression map (Fig. 4), we found that apoptosis-related proteins highly overlap with the key pivotal genes we screened, TNF, IL6, and CASP3. This also verified our conjecture.

**Table 2:** The top 10 Gene Ontology (GO) enrichment items

ID	Term	Category
GO:0019221	cytokine-mediated signaling pathway	biological_process
GO:0043066	negative regulation of apoptotic process	biological_process
GO:0007568	aging	biological_process
GO:0097192	extrinsic apoptotic signaling pathway in absence of ligand	biological_process
GO:0042493	response to drug	biological_process
GO:0045893	positive regulation of transcription, DNA-templated	biological_process
GO:0010628	positive regulation of gene expression	biological_process
GO:1902042	negative regulation of extrinsic apoptotic signaling pathway via death domain receptors	biological_process
GO:0006915	apoptotic process	biological_process
GO:0045944	positive regulation of transcription by RNA polymerase II	biological_process
GO:0032991	protein-containing complex	cellular_component
GO:0005829	cytosol	cellular_component
GO:0005737	cytoplasm	cellular_component
GO:0005615	extracellular space	cellular_component
GO:0097136	Bcl-2 family protein complex	cellular_component
GO:0005654	nucleoplasm	cellular_component
GO:0005576	extracellular region	cellular_component
GO:0000307	cyclin-dependent protein kinase holoenzyme complex	cellular_component
GO:0045121	membrane raft	cellular_component
GO:0005901	caveola	cellular_component
GO:0019901	protein kinase binding	molecular_function
GO:0005515	protein binding	molecular_function
GO:0019899	enzyme binding	molecular_function
GO:0042802	identical protein binding	molecular_function
GO:0008134	transcription factor binding	molecular_function
GO:0042803	protein homodimerization activity	molecular_function
GO:0044877	protein-containing complex binding	molecular_function
GO:0004879	nuclear receptor activity	molecular_function
GO:0000976	transcription regulatory region sequence-specific DNA binding	molecular_function
GO:0003700	DNA-binding transcription factor activity	molecular_function

**Table 3:** The top 20 KEGG enrichment items

ID	Term	*p*-value
hsa04933	AGE-RAGE signaling pathway in diabetic complications	2.51E-30
hsa05200	Pathways in cancer	1.46E-26
hsa05212	Pancreatic cancer	3.22E-20
hsa04210	Apoptosis	1.95E-19
hsa04932	Non-alcoholic fatty liver disease	1.05E-18
hsa05161	Hepatitis B	8.49E-18
hsa05167	Kaposi sarcoma-associated herpesvirus infection	2.41E-17
hsa04931	Insulin resistance	2.78E-17
hsa05160	Hepatitis C	9.24E-17
hsa05215	Prostate cancer	9.57E-17
hsa05162	Measles	1.56E-16
hsa05210	Colorectal cancer	3.07E-16
hsa05163	Human cytomegalovirus infection	6.60E-16
hsa01521	EGFR tyrosine kinase inhibitor resistance	2.05E-15
hsa05206	MicroRNAs in cancer	2.57E-15
hsa04068	FoxO signaling pathway	1.80E-14
hsa04668	TNF signaling pathway	2.44E-14
hsa05418	Fluid shear stress and atherosclerosis	3.86E-14
hsa05164	Influenza A	1.16E-13
hsa04920	Adipocytokine signaling pathway	1.97E-13

**Table 4:** The top 20 Rectome enrichment items

ID	Pathway	Enrichment_score
R-HSA-6785807	Interleukin-4 and Interleukin-13 signaling	7.99E-26
R-HSA-449147	Signaling by Interleukins	1.78E-22
R-HSA-1280215	Cytokine Signaling in Immune system	9.26E-21
R-HSA-168256	Immune System	1.51E-15
R-HSA-5357801	Programmed Cell Death	2.85E-15
R-HSA-109581	Apoptosis	3.89E-14
R-HSA-162582	Signal Transduction	5.31E-14
R-HSA-109606	Intrinsic Pathway for Apoptosis	3.26E-13
R-HSA-6783783	Interleukin-10 signaling	2.12E-12
R-HSA-9006934	Signaling by Receptor Tyrosine Kinases	5.09E-11
R-HSA-5663202	Diseases of signal transduction by growth factor receptors and second messengers	7.04E-10
R-HSA-1643685	Disease	1.13E-09
R-HSA-212436	Generic Transcription Pathway	7.37E-09
R-HSA-2219528	PI3K/AKT Signaling in Cancer	7.43E-09
R-HSA-9009391	Extra-nuclear estrogen signaling	7.69E-09
R-HSA-5218859	Regulated Necrosis	1.90E-08
R-HSA-9634638	Estrogen-dependent nuclear events downstream of ESR-membrane signaling	2.35E-08
R-HSA-982772	Growth hormone receptor signaling	2.35E-08
R-HSA-73857	RNA Polymerase II Transcription	6.51E-08

### Results of molecular docking

**Fig. 5:**
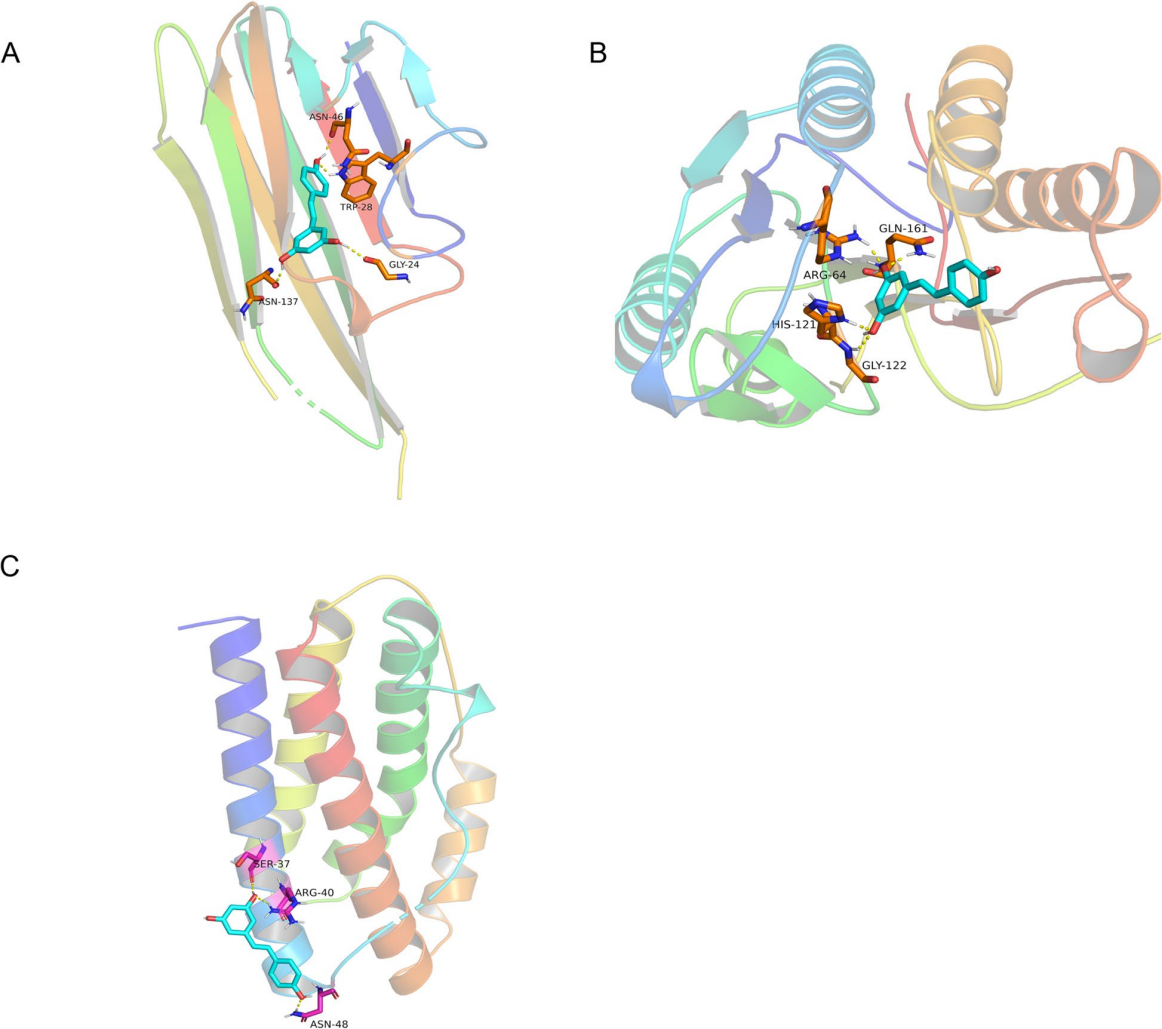
**A** Display of the results pattern diagram for Res docking with TNF, **B** Display of the results pattern diagram for Res docking with CASP3, **C** Display of the results pattern diagram for Res docking with IL6

Combining the results of PPI screening and enrichment analysis, we performed molecular docking of three hub genes of apoptosis (TNF, IL6, CASP3) with Res. The small molecule ligands can spontaneously bind to the protein receptors when the binding energy is <0 kJ mol. If the binding energy is <-5.0 kJ mol or lower, it indicates a better binding ability of both. Three docking results were generated by docking simulations. Among them, the results of TNF-a and Res docking were -6.10, IL6 and Res docking were -4.90, and CASP3 and Res docking were -6.33. They all have binding energies < 0 kJ mol, which means that they all bind well. This molecular docking result indicates that Res can bind to apoptosis key proteins adequately, which may be an important basis for Res regulation of apoptosis treatment OP. Finally, the docking results were visualized with Pymol software (Fig. 5).

### Res inhibits apoptosis to promote osteogenic differentiation in vitro results

#### Res promotes proliferation of MC3T3-E1 cells

**Fig. 6:**
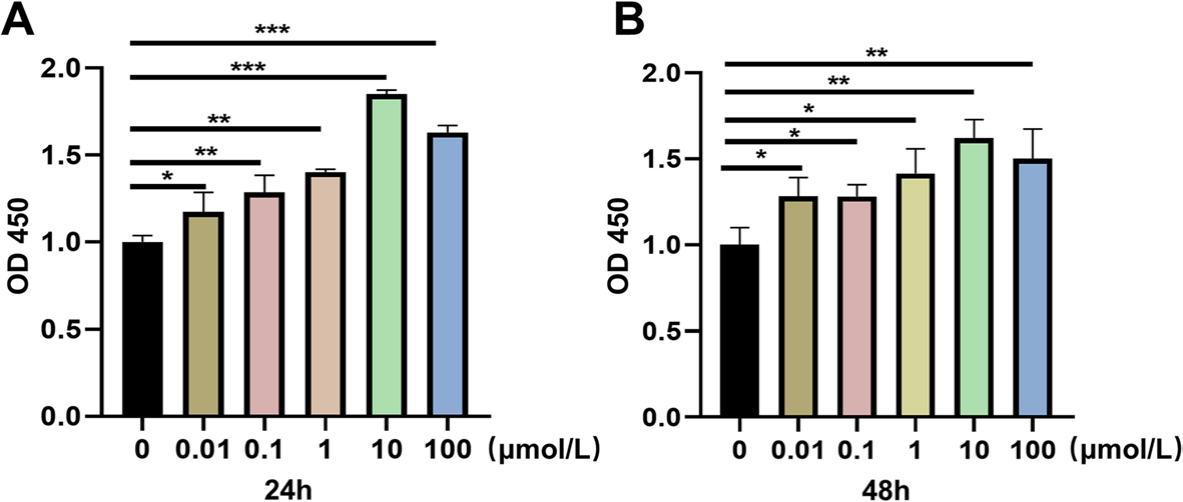
Res promotes the proliferation of MC3T3-E1 cells. **A** MC3T3-E1 cells were treated with 0, 0.01,0.1,1,10 and 100 μmolL Res for 24 h, and cell proliferation was detected by CCK-8. **B** MC3T3-E1 cells were treated with Res for 48 h, and cell proliferation was detected by CCK-8.*P<M0.05, **P<0.01, ***P<0.001 vs control

As demonstrated in Fig. 6, following 0.01, 0.1, 1, 10, and 100 mol/L Res treatment intervention on MC3T3-E1 cells for 24 and 48 h, the proliferation ability of MC3T3-EI cells was increased compared to the blank control group (Supplementary. Table S9-S10). The most noticeable effect was detected at 10 mol/L, but inhibition of value-added occurred at doses up to 100 mol/L (*P*<0.05), demonstrating that Res can promote osteoblast growth at optimum concentrations.

#### Res promotes osteogenic differentiation of MC3T3-E1 cells

**Fig. 7:**
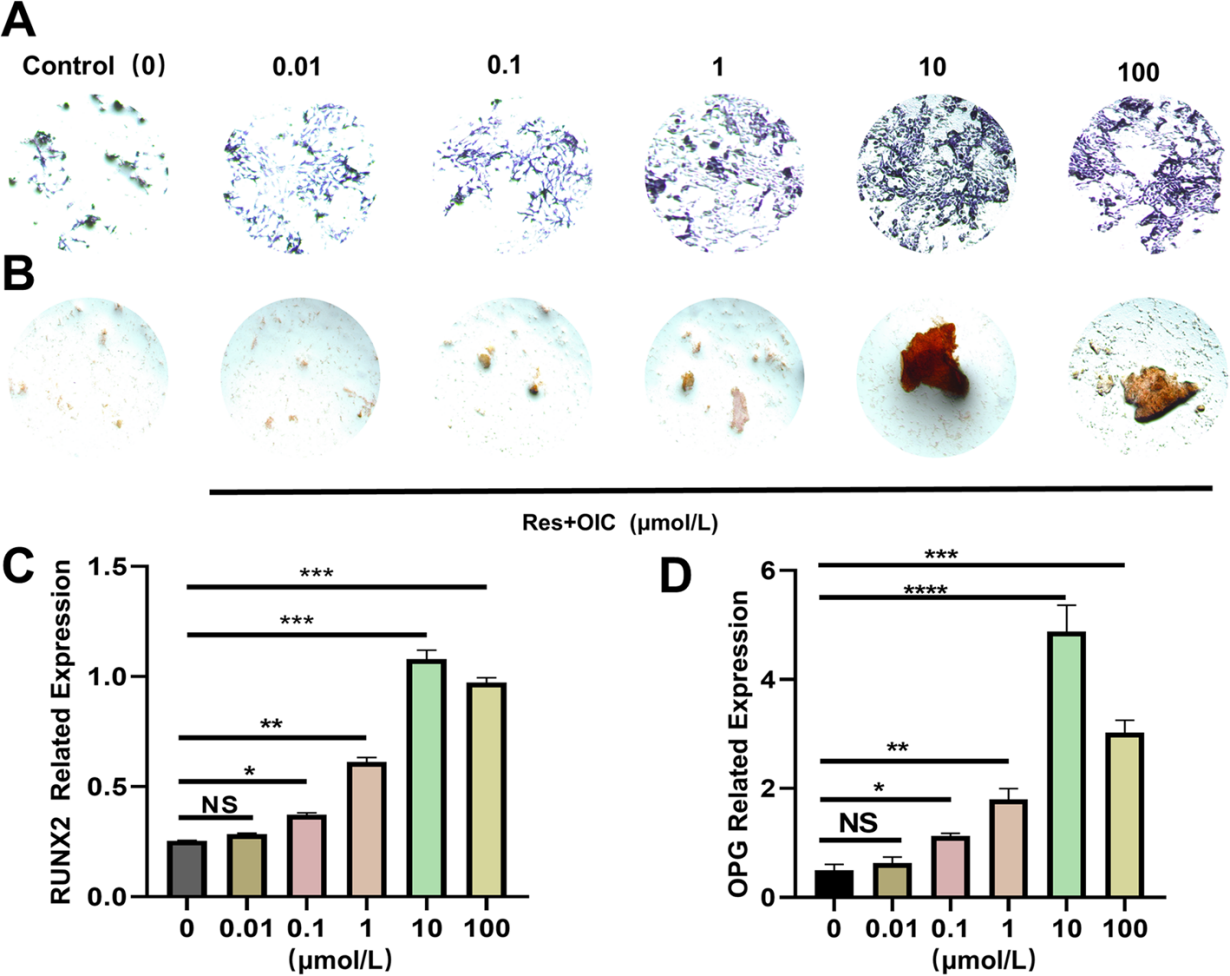
Res promotes osteogenic differentiation of MC3T3-E1 cells. **A** ALP staining in each group (x100). Compared with the control group, the staining degree of 10μmolL Res was the most obvious. **B** The alizarin red staining of each group(x100). Compared with the control group, Mineralized nodules are the most obvious in the 10μmolL Res group. **C** The relative expression of Runx2 mRNA levels. **D** The relative expression of OPG mRNA levels. *P<0.05, **P<0.01,***P<0.001vs control

ALP is an early indication of osteogenic differentiation, and the higher the ALP activity, the more advanced the differentiation. Figure 7A depicts the results of ALP staining after 7 days of osteogenic induction solution incubation: the degree of ALP staining rose as the Res concentration gradually increased. The staining effect was particularly noticeable in the 10 mol/L Res-treated group as compared to the blank control group. The results of alizarin red staining after 21 days of osteogenic induction solution culture are shown in Fig. 7B. The formation of mineralized nodules gradually increased with the concentration of Res, and the mineralization staining was most obvious in the group treated with 10 mol/L Res. Mineralization of extracellular matrix is one of the important signs of osteoblast differentiation and maturation. Res was shown to be able to encourage the development of calcium nodules in MC3T3-EI osteoblasts and, thus, enhance It was encouraged to differentiate osteogenically.

Runx2, a transcription factor downstream of many osteogenic-related pathways, is also important for osteogenic differentiation at an early stage, and its reduction leads to reduced levels of osteogenic differentiation. OPG is a component of the bone extracellular matrix and is one of the main indicators of osteoblast differentiation and maturation into the mineralization phase, which is considered a late marker of osteoblast differentiation and maturation. Q-PCR results are shown in Fig. 7C and D: compared with the control group, the difference between 0.01 μmol/L Res treatment group was not statistically significant (*P*>0.05). The expression levels of Runx2 gene in 0.1, 1 and 10 μmol/L Res groups were all The expression level of Runx2 gene was significantly enhanced in 0.1, 1 and 10 μmol/L Res groups (*P*<0.01), and the expression level of 10 μmol/L treatment group was the most significant. 10 μmol/L Res group could significantly increase the expression level of OPG gene (*P*<0.01), and the difference was statistically significant. This result further indicated that Res could promote osteogenic differentiation of MC3T3-E1 cells.

#### Res promotes MC3T3-E1 proliferation and differentiation through inhibition of apoptosis

**Fig. 8:**
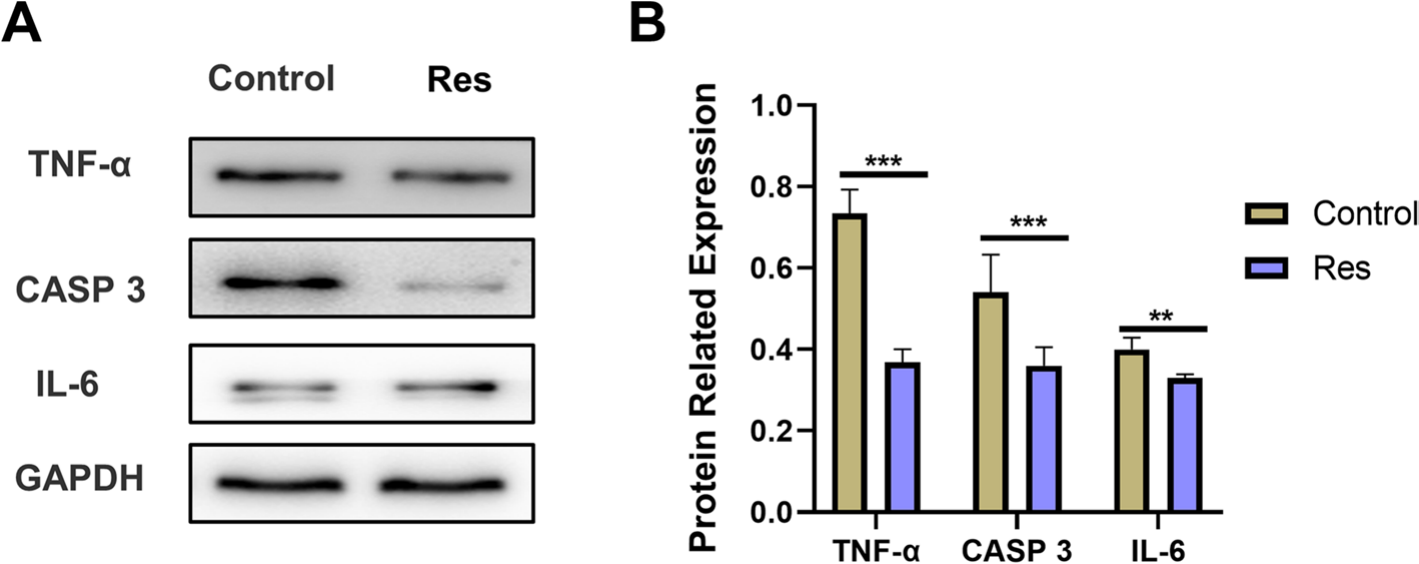
Res promotes the proliferation and differentiation of MC3T3-E1 by inhibiting apoptosis. **A** The electrophoresis of protin expresson. **B** The relative protein expression levels. (Expression level of target protein/GAPDH). *P<0.05, **P<0.01,***P<0.001vs control

Through bioinformatic data analysis, we clarified that apoptosis plays an important role in Res treatment of osteoporosis, and MC3T3-E1 is an important cell in regulating osteoporosis. Therefore, we hypothesized that Res promotes MC3T3-E1 proliferation and differentiation by regulating key targets of apoptosis such as TNF-α, CASP3, and IL-6. The expression of apoptosis-related marker proteins TNF-α, CASP 3 and IL-6 was detected by Western blot assay as in Fig. 8 (Supplementary. Table S7). Compared with the control group, Res significantly reduced the expression of TNF-α, CASP 3 and IL-6 (*P*<0.05), which indicated that Res could inhibit the apoptosis level of MC3T3-E1 and thus promote the proliferation and differentiation of MC3T3-E1.

## Discussion

OP is a metabolic bone disease where bone resorption by osteoclasts is more active than bone synthesis by osteoblasts [40]. Because of lack of estrogen, postmenopausal women have more prominent imbalance between osteogenesis and osteolysis [41]. Res has been used to treat postmenopausal OP patients as an estrogen replacement therapy [41]. Research indicates that Res encourages osteoblast differentiation [42]. However, the fundamental processes of Res for postmenopausal, are not fully known. The relationships between disease, drug, and target can be partially accounted for by network pharmacology as a method of drug development [42, 43]. As a result, using network pharmacology and bioinformatics, we investigated the molecular mechanism of Res for the treatment of OP. In vitro tests were also used to confirm the analysis's findings. This research advances our knowledge of the molecular basis of Res' ability to cure OP, which is crucial for future therapeutic applications.

Our study initially investigated the molecular mechanism of Res promoting osteogenesis to improve osteoporosis based on network pharmacological. In addition, we verified the potential mechanism of resveratrol promoting osteogenesis several times by molecular docking and cellular experiments. Based on the bioinformatics data analysis, 91 intersectional targets of Res for OP were initially screened, and 26 key targets of Res for OP were further screened and obtained. The top 10 targets playing important roles were: AKT1, IL6, IL1B, VEGFA, CASP3, STAT3, MAPK3, MYC, PPARG, CTNNB1. A combined GO, KEGG and Reactome biological data analysis was performed based on 91 potential therapeutic targets. GO analysis showed that the regulatory role of biological processes in Res therapeutic OP mainly acted through cytokine-mediated signaling pathways, negative regulation of apoptotic processes, and senescence. KEGG data showed that the biological process of Res treatment of OP acts mainly through apoptosis, TNF signaling pathway, etc. In addition, based on Reactome analysis, the molecular mechanism of Res treatment of OP also includes the regulation of interleukin-like related inflammatory factors, apoptosis. Both KEGG and Reactome analyses suggested that the molecular mechanism of Res treatment of OP involved apoptosis, and the apoptosis pattern map of KEGG database showed that key apoptotic proteins such as TNF, IL6 and CASP3 played important roles, which was consistent with the results of the current analysis. Molecular docking simulations further validated the binding ability between TNF-a, IL6 and CASP3 target proteins and Res, confirming the importance of the aforementioned targets in Res therapeutic OP.

We examined the impact of Res on the proliferation, differentiation, and associated protein expression of pre-osteoblastic MC3T3-E1 cells in order to further confirm the regulatory mechanism of Res on OP. First, we showed through the CCK8 experiment that Res can increase the value-added of MC3T3-EI cells. Additionally, Liu Xiang findings' proved that Res can increase the proliferation of MC3T3-E1 osteoblasts by activating autophagy through GATA-1. In a previous study, it was discovered that Res can increase resistance to oxidative damage by activating the SIRT1/FoxO1 signaling pathway, hence increasing osteogenesis. This finding offered a fresh perspective on the prevention and treatment of osteoporosis [43]. In the current study, our results revealed that Res boosted osteoblast differentiation, and the findings of the ALP and alizarin red staining partially supported our theory, showing that the Res-treated group had a higher rate of mineralized nodule formation. We further confirmed that Res encourages the development of calcium nodules in MC3T3-EI osteoblasts and, thus, encourages osteogenic differentiation.

Apoptosis accelerates the death of osteoblasts as an organism ages, making it a significant contributor to osteoporosis [3]. By using bioinformatic analysis, we discovered that apoptotic expression occurs during osteoblast differentiation and that Res can prevent apoptosis in related cells. Therefore, we hypothesize that Res encourages the osteogenic differentiation of MC3T3-E1 cells and regulates it, which may potentially be connected to the prevention of apoptotic expression. We discovered that Res inhibited the expression of the key apoptosis proteins TNF, IL6, and CASP3, and that the expression of apoptotic proteins was lowest in the 10 mol/L Res group, indicating that Res could inhibit MC3T3-E1 cell apoptosis and promote the regulation of osteogenic differentiation and value-added differentiation, which ultimately effectively improved OP.

## Conclusion

In conclusion, bioinformatics enabled us to discover that Res therapeutic OP involved in cytokine-mediated signaling pathway, negative regulation of apoptotic process. Particular, Apoptosis, FoxO signaling pathway, and TNF signaling pathway are the primary KEGG signaling pathways, Res regulates apoptosis-related proteins TNF, IL6, and CASP3, suppresses osteoblast apoptosis, stimulates osteoclastogenesis, and cures OP. Additional tests supported the hypothesis that Res can reduce MC3T3-E1 cell death and increase the development of these cells into osteoblasts, hence enhancing osteoblast differentiation and bone production. Despite the computational errors in the results of this analysis via online data, and the experimental design is not perfect, these results offer a fresh approach to the management of postmenopausal OP. We will further discover the molecular mechanism of Res for OP through clinical data and experimental validation.

### Supplementary Information

**Supplementary Material 1.**

**Supplementary Material 2.**

## Data Availability

Raw data has been made available. All data generated or analysed during this study are included in this published article and its supplementary information files.

## Abbreviations

Res: Resveratrol
OP: Osteoporosis
TCMSP: Traditional Chinese Medicine Systematic Pharmacology
GO: Gene Ontology
KEGG: Kyoto Encyclopedia of Genes and Genomes
CCK-8: Cell Counting Kit-8
ALP: Alkaline phosphatase staining
BP: Biological process
CC: Cellular components
MF: Molecular function
TNF: Tumor necrosis factor
IL6: Interleukin-6
CASP3: Caspase 3
