# Supplementary material for: Molecular mechanism of resveratrol promoting differentiation of preosteoblastic MC3T3-E1 cells based on network pharmacology and experimental validation
Source: BMC Complement Med Ther. 2024 Feb 29;24:108. doi: 10.1186/s12906-024-04396-3 (PMC10905894; doi:10.1186/s12906-024-04396-3)

Our strips are, from left to right, the control and resveratrol-treated groups. One strip was replicated twice.The red boxes mark the groups we use in Figure 8.All of our images have been exposed with only one development.

Supplement figure for Figure 8：

TNF-α Control group, Res group





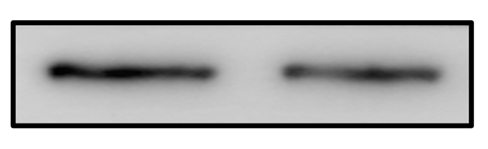




CASP3 Control group, Res group







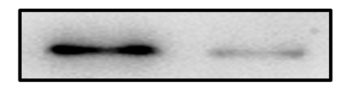


IL6 Control group, Res group


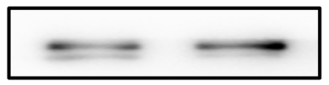




GAPDH Control group, Res group


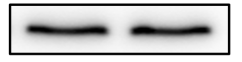

Supplement: Supplementary file 2 — Supplementary Material 2. [file 12906_2024_4396_MOESM2_ESM.doc]
